# The association between reproductive history and menopausal symptoms: an evidence from the cross-sectional survey

**DOI:** 10.1186/s12905-022-01715-z

**Published:** 2022-04-27

**Authors:** Seyedeh Hajar Sharami, Roya Faraji Darkhaneh, Nasrin Ghanami Gashti, Mandana Mansour-Ghanaei, Sedighe Bab Eghbal

**Affiliations:** Reproductive Health Research Center, Department of Obstetrics and Gynecology, Al-Zahra Hospital, School of Medicine, Guilan University of Medical Sciences, P.O. Box: 4144654839, Rasht, Iran

**Keywords:** Menopause, Menopausal symptoms, Reproductive history, Menopause Rating Scale

## Abstract

**Background:**

During menopause, women experience annoying symptoms which may affect their daily activities and quality of life. This study aimed to determine whether reproductive history, an important indicator of estrogen exposure across the lifetime, is associated with the severity of menopausal symptoms in women.

**Methods:**

This study was a cross-sectional study conducted on 214 women aged 35–65 who were randomly selected, and data was collected by a predesigned structured questionnaire. Each item was graded by subjects and a total score was obtained by summing all subscale scores.

**Results:**

There was a significant association between the somatic, psychological, and urogenital menopausal symptoms and reproductive characteristics. Women with a history of abortion had greater total (β = 0.194, *p* = 0.009), and psychological (β = 0.230, *p* = 0.002) symptoms score. Women with higher number of children were more likely to have higher somatic (β = 0.212, *p* = 0.005) symptoms than others.

**Conclusions:**

Our findings showed reproductive factors may have an influence on the severity of menopausal symptoms. After confirmation by further studies, these findings may help target women at risk of more severe menopausal symptoms at later ages.

**Supplementary Information:**

The online version contains supplementary material available at 10.1186/s12905-022-01715-z.

## Background

Menopause is a condition caused by the depletion of ovarian function and then amenorrhea for at least 12 months after the last menstrual period without pathologic or surgical causes [1]. During menopause, women often experience some annoying symptoms which may affect their daily activities and quality of life. Menopausal symptoms vary according to some conditions such as racial groups [2]. Menopausal health problems present a significant public health issue requiring specific programs and management. The etiology of symptoms experienced by women at menopause is multifactorial [3].

Women are exposed to reproductive factors during their life that are associated with a variety of hormonal and metabolic changes, which could impact their health. The symptoms of menopause are caused by a decline in estrogen production in the presence of continued production of gonadotrophic hormones resulting in bone loss, urogenital atrophy, urinary tract infections, incontinence, increased cardiovascular risk, sexual dysfunction, and loss of skin elasticity [4]. Some of the risk factors for menopausal symptoms are the presence of chronic diseases, symptoms of anxiety, depression [5], and a low level of physical exercise [6].

The menopausal transition coincides with the onset of aging, and there is a significant overlap between symptoms occurring with aging and menopause. This is a problem for health care providers to determine whether a specific symptom is related to the ovarian hormonal changes during menopause or to the aging process [7]. By including participants in their early 40 s who were still menstruating, it has been demonstrated that vasomotor symptoms (hot flashes and night sweats), sleep disruption, and vaginal dryness are mostly related to a loss of estrogen, and most other symptoms have at least a significant component related to aging [8].

Reproductive history has been found as an involved factor in menopausal symptoms. It has been demonstrated that early age at menopause and higher parity have a deleterious effect on the motor function that persists in older people [9]. It has been reported in a systematic review and meta-analysis that previous use of oral contraceptives, age at menarche ≥ 13, and having at least one live birth are associated with later menopause. These results suggest that the aforementioned reproductive factors could be markers of later ovarian aging [10].

Bjelland et al., (2018) found that age at menarche over 15 years slightly decreased the risk of natural menopause between 1 and 5%, however, the same study found that the time between menarche and menopause, was 9 times higher in women with age at menarche ≤ 9 years compared to age at menarche ≥ 17 years [11]. An increased risk of earlier natural menopause among nulliparous versus parous women has been demonstrated, and a delay in menopause with an increase in the number of live births [12, 13]. Sharma et al. reported that age at menopause is significantly correlated with age at menarche and maternal age at birth of first and last child, but not with menopausal symptom rating scores [14]. Menopausal symptoms are very complicated in terms of experience, severity, and dynamics [15, 16].

The frequency and severity of menopausal symptoms are variable and associated with multiple physical, vasomotor, psychological, and sexual complaints. It has been demonstrated that reproductive factors were significantly associated with the risk of depressive symptoms in postmenopausal women [17]. The BMI and lifestyle are modifiable conditions and change of them may improve menopausal complications. It has been reported that vasomotor symptoms and sleep problems can be dominant in the face of high FSH [18]. Early age at menarche is also a risk factor for vasomotor symptoms, but midlife BMI may play an important role in modifying this risk [19].

The health issues and life quality of post-menopausal women have become a growing concern in the healthcare systems worldwide. Since some of the risk factors affecting menopausal symptoms are race/ethnicity [2], stress, nutrition, and BMI, these factors may be different in the Iranian population. To the best of our knowledge, the association between reproductive factors and the severity of menopausal symptoms in North Iranian women has not been evaluated. Therefore, the present study aimed to determine whether reproductive history, an important indicator of estrogen exposure across the lifetime, is associated with the severity of menopausal symptoms in women. Our hypothesis was that reproductive history was associated with the severity of menopausal symptoms. Considering that the issues related to menopause are complicated, the results of this study may contribute to an understanding of particular health needs of menopausal women and assist public health managers in devising appropriate prevention or intervention programs for meeting such needs.

## Methods

This study was a cross-sectional study conducted on women visiting the gynecology clinic of Al-Zahra Hospital, Rasht, Iran, from June 2020 to April 2021. The sampling technique was convenience sampling and samples were selected from the population based on their availability to the researcher. The sample size was estimated based on the study of Thapa et al. [20]. Considering the 95% confidence interval and 90% test power, 214 samples were obtained with a 10% dropout rate. From the total of 3801 women aged 35–65 who attended the gynecology clinic during the study period, 294 women were approached, because sampling was done on two days of the week. 294 women were approached, 274 women agreed to participate in the study, and 60 women were ineligible based on inclusion and exclusion criteria. The percentage of approached women who agreed to participate in the study was 92.88%. Eligibility was determined after approaching women to participate (Additional files 1 and 2).

Women with medically or surgically induced menopause, women with early menopause (younger than 45 years) or premature menopause (younger than 40 years), those who used hormone replacement therapy, and pregnant or lactating women were excluded from the study. Also, participants with total hysterectomy, ovarian surgery or chemo/radiotherapy, any major organ disease, had used hormone therapy, with a history of major depressive disorder, schizophrenia, and general psychiatric conditions were excluded. There was no comorbid condition in the study participants. For 214 women who met inclusion criteria and agreed to participate in the study, data was collected by the researchers by face-to-face interview; each interview lasted 10–15 min and was conducted using a predesigned structured questionnaire. Informed consent was taken from all participants before data collection. Before starting the study, ethical approval was obtained from the Research Deputy and Ethics Committee of Guilan University of Medical Sciences (Approval ID: IR.GUMS.REC.1399.395).

The study population was divided into three groups (pre-, peri-, and postmenopausal status) based on the WHO definitions [21]. The menopausal status of participants was determined by a gynecologist based on WHO definition: pre-menopausal women are those who have experienced regular menstrual bleeding within the last 12 months, peri-menopausal women are defined as those women who have experienced irregular menses within the last 12 months or the absence of menstrual bleeding for more than 3 months but less than 12 months, and post-menopausal women are those who have not experienced menstrual bleeding for 12 months or more. Questions and medical history about menopausal status were asked by a gynecologist and were not a part of the questionnaire. The questionnaire was developed in two sections. The first section is a predesigned questionnaire that examines demographic and reproductive factors (age, body mass index (BMI), age of menarche, age of first pregnancy, parity, number of pregnancies, number of abortions, number of deliveries, type of delivery, menopausal status, history of gynecological surgery (surgery for ovarian cyst, surgery for pelvic prolapse, myomectomy, pelvic laparoscopy, and tubal ligation), history of using hormonal contraception, and number of children). The second part of the questionnaire, which was a list of menopausal symptoms, used the valid questionnaire of Menopause Rating Scale (MRS). It consists of eleven items divided into three subscales: somatic, psychological, and urogenital symptoms [22]. The somatic symptoms include hot flushes, heart, sleep, and joint complaints; psychologic symptoms include depression, irritability, anxiety, and exhaustion; and urogenital symptoms include sexual, bladder, and dryness complaints. Each item was graded by subjects from 0 (not present) to 4 (1: mild, 2: moderate, 3: severe, and 4: very severe), and a total score was obtained by summing all subscale scores.

### Statistical analysis

Data were analyzed by Statistical Package for Social Sciences (SPSS) software version 21.0 (SPSS Inc, Chicago, IL, USA). Baseline demographic and clinical data were described by descriptive statistics. Results are presented as number (percentage) and mean ± SD (standard deviation) wherever appropriate. Kruskal–Wallis and Mann–Whitney tests were applied to compare the frequency and severity of symptoms among the pre-, peri-, and postmenopausal groups. Multiple linear regression analysis was used to assess the association between reproductive factors and menopausal symptoms. A *p* value of < 0.05 was considered statistically significant.

## Results

We included 214 women with a mean age of 46.49 ± 8.53 years in this study. Women who were included in the study were not using hormones or were naturally postmenopausal. Demographic and reproductive data of the study group are presented in Table 1. Three groups of the study included 70 premenopausal, 72 perimenopausal, and 72 postmenopausal women. Forty-three percent of the participants were obese (BMI > 30), and there was a significant association between high BMI and somatic symptoms (*p* = 0.045). The mean score of somatic, psychological, and urogenital symptoms reported by participants, and the association of demographic and reproductive factors with the severity of symptoms has been presented in Table 2. The mean score for somatic and urogenital symptoms was higher in postmenopausal women than in other groups. Perimenopausal women had a higher mean of psychological symptoms, however, it was not significantly different from postmenopausal women.

**Table 1:** Frequency and percentage of demographic and reproductive data of the study groups

Characteristics		Menopausal status (n, %)			*p* value
		Premenopause	Perimenopause	Postmenopause	
Age	$\overline{X}$ ± S.D	41.24 ± 5.31	42.51 ± 5.35	55.57 ± 5.93	0.001*
BMI	$\overline{X}$ ± S.D	29.11 ± 4.56	31.76 ± 5.45	28.99 ± 4.77	0.001*
Age of menarche	$\overline{X}$ ± S.D	13.07 ± 1.66	13.03 ± 2.01	12.87 ± 1.67	0.59**
Age of first pregnancy	$\overline{X}$ ± S.D	22.63 ± 5.29	20.35 ± 3.57	21.64 ± 5.12	0.132**
Parity	$\overline{X}$ ± S.D	1.27 ± 1.08	1.67 ± 1.18	3.44 ± 2.13	0.001*
Number of abortions	$\overline{X}$ ± S.D	0.33 ± 0.67	0.44 ± 0.81	0.55 ± 0.95	0.387*
Number of children	0	23 (32.9%)	20 (27.8%)	32 (44.4%)	0.018*
	1	29 (41.4%)	23 (31.9%)	17 (23.6%)	
	2	18 (25.7%)	22 (30.6%)	17 (23.6%)	
	3	0 (0.0%)	7 (9.7%)	6 (8.3%)	
Age of menopause	$\overline{X}$ ± S.D	–	–	47.68 ± 5.57	–
Type of delivery	No delivery	20 (28.6%)	18 (25%)	2 (2.8%)	0.001*
	Cesarean section (CS)	28 (40%)	23 (31.9%)	11 (15.3%)	
	Natural childbirth (NC)	18 (25.7%)	23 (31.9%)	45 (62.5%)	
	CS + NC	4 (5.7%)	8 (11.1%)	14 (19.4%)	
Gynecological surgery history	Yes	35 (50%)	47 (65.3%)	41 (56.9%)	0.195**
	No	35 (50%)	25 (34.7%)	31 (43.1%)	
History of using hormonal contraception	Yes	33 (47.1%)	39 (54.2%)	46 (63.9%)	0.134**
	No	37 (52.9%)	33 (45.8%)	26 (36.1%)	

BMI: body mass index; $\overline{X}$ ± S.D: Mean ± Standard deviation. The *p* value is a test for a difference between the mean of continuous variables, and for a difference between the categorical variables. The asterisk by the *p* values indicates which tests are ''*Kruskal-Wallis'' or ''**Mann-Whitney''

**Table 2:** Association of demographic and reproductive factors with the severity of menopausal symptoms. Total scores that represent the severity of symptoms were obtained by summing all subscale scores

Characteristics		Somatic symptoms	Psychological symptoms	Urogenital symptoms	Total
Menopausal status	Premenopausal	11.17 ± 5.01	19.97 ± 8.48	9.87 ± 4.01	46.96 ± 14.51
	Perimenopausal	20.61 ± 6.02	23.21 ± 7.64	10.67 ± 3.58	54.48 ± 13.53
	postmenopausal	21.43 ± 6.38	23.05 ± 8.69	13.68 ± 3.96	58.17 ± 15.55
	*p* value*	0.001	0.004	0.001	0.001
Age	Below 40	18.11 ± 4.81	22.26 ± 8.58	10.83 ± 3.89	51.20 ± 14.40
	40–50	19.97 ± 6.77	22.51 ± 8.44	10.02 ± 4.01	52.50 ± 16.02
	Over 50	20.98 ± 6.10	21.46 ± 8.20	13.61 ± 3.74	56.06 ± 14.78
	*p* value*	0.032	0.687	0.001	0.132
BMI	Below 25	18.84 ± 5.54	20.09 ± 7.63	12.19 ± 4.06	51.12 ± 13.86
	25–30	18.89 ± 6.10	21.56 ± 8.03	11.44 ± 4.34	51.89 ± 15.18
	Over 30	20.89 ± 6.17	23.33 ± 8.84	11.13 ± 4.05	55.35 ± 15.59
	*p* value*	0.045	0.139	0.425	0.174
Age of menarche	Below 13	19.92 ± 6.13	21.59 ± 7.78	11.58 ± 4.19	53.09 ± 14.83
	Over 13	19.39 ± 6.09	23.09 ± 9.42	11.09 ± 4.14	53.58 ± 16.06
	*p* value**	0.588	0.494	0.421	0.977
Age of first pregnancy	Below 20	21.33 ± 5.86	23.42 ± 8.16	12.33 ± 4.24	57.09 ± 14.47
	Over 20	19.57 ± 6.20	22.20 ± 9.04	11.67 ± 4.23	53.45 ± 15.40
	*p* value**	0.025	0.18	0.342	0.042
Parity	Nulliparous	16.30 ± 5.03	18.57 ± 6.46	8.65 ± 2.53	43.53 ± 12.51
	1–3	20.37 ± 5.97	22.64 ± 8.64	11.58 ± 4.17	54.59 ± 14.87
	4–7	21.25 ± 6.55	24.09 ± 8.29	14.19 ± 3.82	59.53 ± 14.75
	*p* value*	0.001	0.001	0.001	0.001
Number of abortions	No	18.73 ± 5.55	21.54 ± 8.04	11.19 ± 4.20	51.46 ± 14.66
	1–2	22.25 ± 6.60	23.27 ± 8.68	11.83 ± 3.87	57.35 ± 15.11
	3–4	21.73 ± 7.55	24.27 ± 11.12	12.64 ± 5.12	58.64 ± 19.38
	*p* value*	0.002	0.378	0.406	0.028
Number of children	0	18.53 ± 5.97	19.48 ± 7.01	10.61 ± 4.09	48.63 ± 14.30
	1	20.17 ± 5.73	22.62 ± 8.79	11.78 ± 4.18	54.58 ± 14.62
	2	20.28 ± 6.51	23.96 ± 8.69	12.05 ± 4.24	56.29 ± 16.24
	3	22.08 ± 6.45	26.23 ± 8.49	11.38 ± 3.99	59.69 ± 13.17
	*p* value*	0.111	0.002	0.140	0.004
Type of delivery	No delivery	16.30 ± 5.03	18.57 ± 6.46	8.65 ± 2.53	43.53 ± 12.51
	Cesarean section (CS)	19.44 ± 5.05	22.55 ± 8.25	11.16 ± 4.05	53.14 ± 13.32
	Natural childbirth (NC)	20.62 ± 6.35	22.92 ± 8.94	12.53 ± 4.48	56.07 ± 15.82
	CS + NC	22.88 ± 6.85	23.73 ± 8.34	12.61 ± 3.42	59.23 ± 15.12
	*p* value*	0.001	0.013	0.001	0.001
Gynecological surgery history	Yes	20.45 ± 6.19	22.32 ± 7.82	11.60 ± 4.08	54.36 ± 14.33
	No	18.79 ± 5.89	21.80 ± 9.12	11.17 ± 4.30	51.77 ± 16.30
	*p* value**	0.039	0.232	0.369	0.087
History of using hormonal contraception	Yes	20.25 ± 5.89	22.82 ± 8.71	12.09 ± 4.44	55.17 ± 14.76
	No	19.11 ± 6.33	21.21 ± 7.90	10.59 ± 3.68	50.92 ± 15.52
	*p* value**	0.086	0.211	0.017	0.023

BMI: body mass index; data has been shown in form of $\overline{X}$ ± S.D (Mean ± Standard deviation). The *p* value is a test for a difference between the mean of continuous variables, and for a difference between the categorical variables. The asterisk by the *p* values indicates which tests are ''*Kruskal-Wallis'' or ''**Mann-Whitney''

A significant association was found between the three types of menopausal symptoms and gravidity (*p* < 0.001), parity (*p* < 0.001), and type of delivery (*p* < 0.001). There was a significant association between the age of first pregnancy and history of gynecological surgery with somatic symptoms (*p* = 0.025, and *p* = 0.039, respectively). History of hormonal contraceptive consumption was significantly correlated to urogenital symptoms (*p* = 0.017). The number of abortions was significantly correlated to somatic symptoms (*p* = 0.002). Interestingly, a higher number of children was significantly associated with psychological symptoms (*p* = 0.002). There was no significant correlation between the menopausal symptoms with the age of menarche (*p* = 0.977).

Multiple linear regression analysis of the association between reproductive factors and menopausal symptoms are shown in Table 3. Variables that were significantly associated with the severity of menopausal symptoms (Table 2) were included in the regression model, then non-significant variables were removed in the multiple regression step by the Backward regression process (Additional files 1, 2). Age effect as confounding variable was controlled. Women with a history of abortion had greater total (β = 0.194, *p* = 0.009), and psychological (β = 0.230, *p* = 0.002) symptoms score. Women with higher number of children were more likely to have higher somatic (β = 0.212, *p* = 0.005) symptoms than others. Higher age of first pregnancy was associated with lower psychological symptoms (β = − 0.191, *p* = 0.010). Based on regression models, no other reproductive factor was significantly associated with the severity of menopausal symptoms.

**Table 3:** Multiple linear regression analysis of the association between reproductive factors and menopausal symptoms

Variables	Model of Total		
	β	t	*p*
Number of abortions	0.194	2.633	0.009
Number of children	0.131	1.782	0.077
Constant		17.125	< 0.001
Adj R^2^	0.064		
F	4.943		
P	0.003		

Variables	Model of psychological		
	β	t	*p*
Number of abortions	0.230	3.156	0.002
Age of first pregnancy	− 0.191	− 2.616	0.010
Constant		12.198	< 0.001
Adj R^2^	0.075		
F	8.061		
P	< 0.001		

Variables	Model of somatic		
	β	t	*p*
Number of abortions	0.141	1.895	0.060
Number of children	0.212	2.854	0.005
Constant		16.299	< 0.001
Adj R^2^	0.048		
F	5.377		
P	0.005		

No reproductive factor was associated with severity of menopausal symptoms in model of urogenital

Effect of age as confounding variable was controlled. Using multiple linear regression and using the Backward model all significant variables from Table 2 that were effective on menopausal symptoms were included in the regression model, then non-significant variables were removed in the multiple regression steps by the Backward regression process. Age at first pregnancy for women who were never pregnant was defined with replacing missing values method, with the mean of Age at first pregnancy for women who experienced pregnancy

## Discussion

This study evaluated the frequency and severity of menopausal symptoms and their association with reproductive factors among North Iranian women. Women who were included in the study were not using hormones and were naturally postmenopausal. The results of our study showed a significant association between the somatic, psychological, and urogenital menopausal symptoms and reproductive characteristics. Women with a history of abortion had greater total, somatic, and psychological symptoms score. Women with a higher number of children were more likely to have higher total and somatic symptoms than others. Many organs of the human body are sensitive to estrogen through chemical, biochemical, and genomic mechanisms [23], so a reduction in estrogen level in menopause gives rise to a number of physical, psychological, and sexual changes [24]. The frequency of menopausal symptoms varies over time. Some symptoms decrease over time while others increase progressively and become more severe over time [15, 25].

Similar to previous studies [20, 26], the present study found a lower frequency of urogenital symptoms than somatic and psychological symptoms in three menopause status groups. The severity of symptoms was lowest in the premenopausal group and highest in the postmenopausal group. In the present study, almost all of the studied reproductive characteristics except the age of menarche were significantly associated with at least one of the three somatic, psychological, and urogenital symptoms. Age of first pregnancy (below 20) and the number of abortions were significantly associated with the severity of somatic symptoms. Women with higher parity and a history of any type of delivery had more severe somatic, psychological, and urogenital symptoms. History of using hormonal contraception was significantly associated with urogenital symptoms. In our study, higher parity and history of abortion were significantly associated with menopausal symptom severity, which is consistent with the results of other studies [20, 27].

Results of the present study showed women's reproductive history has significant effects on the severity of menopausal symptoms. Participants who have a high number of children might be under more stress and pressure, which may put them at risk of more severe symptoms [20]. It has been reported women with three or more children were at higher risk of disability in advanced age compared to nulliparous women [9]. Nulliparity is also associated with an early onset of menopause as parous women have an anovulatory period that delays menopause [28]. It has been reported that postmenopausal women with three or more children had worse physical function than nulliparous women [29].

Our finding showed that higher BMI was significantly associated with the severity of somatic symptoms. In previous studies, higher BMI was found to be associated with an increasing number of symptoms [30, 31]. Obese women have higher levels of estrogen due to greater peripheral conversion of androgens to estrogens in adipose tissue. However, it is assumed that adipose tissue, while forming estrogens from circulating androgen precursors, also produces hormones/cytokines, for example, leptin, tumor necrosis factor (TNF), that may suppress ovarian steroid production and influence thermoregulation, thus explaining the higher risk and frequency of symptoms such as hot flushes [30].

The level of estrogen exposure is different in women due to different reproductive history and use of estrogen-containing drugs, and endogenous estrogen exposure is highest during a woman's reproductive life. Although the length of the reproductive period is associated with women's lifetime estrogen exposure, reproductive activity during this time also influences endogenous estrogen exposure. Although the estrogen level is high during pregnancy, women with more parity have lower circulating estrogen over lifetime than women with fewer parity or nulliparity [32]. Age at first birth may affect lifetime endogenous estrogen exposure. It has been demonstrated that later age at first birth would indicate longer time spent in a nulliparous state of higher estrogen levels [33], and results in decreased risk of depressive symptoms. Besides endogenous estrogen exposure, exogenous estrogen use and related factors may also modify the risk of depressive symptoms. However, the role of estrogen in elevating symptoms is a possible hypothesis and requires further studies to confirm.

Multiple linear regression analysis showed that women with a history of abortion had greater total, somatic, and psychological symptoms scores. Women with a higher number of children were more likely to have higher total and somatic symptoms. And interestingly higher age of first pregnancy was associated with lower psychological symptoms. These findings may be due to the stress of raising children, which increases with the number of children. Miscarriages or infants die and having more births increase a woman's risk of depressive symptoms in later life [17].

It is important to have a clear categorization of the menopausal process and attribution of symptoms. Based on the Study of Women’s Health Across the Nation (SWAN) [7], race/ethnicity, socio-economic status and adverse life events, and BMI are major factors that influence menopausal symptoms. Some of these factors like BMI and lifestyle are modifiable conditions and change of them may improve menopausal complications and subsequent health and risks for disease. It has been reported that vasomotor symptoms and sleep problems can be dominant in the face of high FSH [18]. In addition, early age at menarche is a risk factor for vasomotor symptoms, but midlife BMI may play an important role in modifying this risk [19].

This study had some limitations. The first limitation was related to the design of the study which was cross-sectional, because the cross-sectional design can determine associations, not causal relationships. Second, the most of the study participants were women who were referred to women's health clinics, and did not include women who did not refer a doctor, so the results of this study may not be generalizable to all women. Third, our study data was limited to only one hospital, so the findings of this study cannot be generalized to all Iranian women. Finally, laboratory data on serum estrogen levels were not available to determine the biological role of estrogen in menopausal symptoms.

## Conclusion

The health issues and life quality of post-menopausal women have become a growing concern in the healthcare systems worldwide. Some of the risk factors affecting menopausal symptoms are ethnicity, stress, nutrition, and BMI, which are different in the different populations. The results of this study may contribute to an understanding of particular health needs of menopausal women and assist public health managers in devising appropriate prevention or intervention programs for meeting such needs. This study showed that reproductive history may have an influence on the severity of menopausal symptoms. These findings may help target women at risk of more severe menopausal symptoms at later ages. If further studies confirm the link between reproductive history and the severity of menopausal symptoms, this information could help healthcare providers to counsel and manage aging women.

## Supplementary Information

**Additional file 1**. Multicollinearity analysis.

**Additional file 2**. Outlier analysis.

## Data Availability

The datasets used and/or analyzed during the current study are available from the corresponding author on reasonable request.

## Abbreviations

WHO: World Health Organization
BMI: Body mass index
MRS: Menopause Rating Scale
SPSS: Statistical Package for Social Sciences
SD: Standard deviation
TNF: Tumor necrosis factor
