# Supplementary material for: The association between reproductive history and menopausal symptoms: an evidence from the cross-sectional survey
Source: BMC Womens Health. 2022 Apr 27;22:136. doi: 10.1186/s12905-022-01715-z (PMC9044690; doi:10.1186/s12905-022-01715-z)
Supplement: Supplementary file 1 — Additional file 1. Multicollinearity analysis. [file 12905_2022_1715_MOESM1_ESM.docx]

Supplementary file 1 : multicollinearity analysis.

| **Coefficients^a^** | | | | | | | | | | | | | |
| --- | --- | --- | --- | --- | --- | --- | --- | --- | --- | --- | --- | --- | --- |
| Model | | Unstandardized Coefficients | | Standardized Coefficients | t | Sig. | 95% Confidence Interval for B | | Correlations | | | Collinearity Statistics | |
|  |  | B | Std. Error | Beta |  |  | Lower Bound | Upper Bound | Zero-order | Partial | Part | Tolerance | VIF |
| 1 | (Constant) | 49.626 | 6.741 |  | 7.361 | .000 | 36.317 | 62.934 |  |  |  |  |  |
|  | Number of children | 2.166 | 1.264 | .127 | 1.714 | .088 | -.329 | 4.660 | .114 | .131 | .126 | .982 | 1.018 |
|  | Number of abortion | 3.581 | 1.344 | .198 | 2.663 | .008 | .926 | 6.235 | .183 | .201 | .196 | .982 | 1.019 |
|  | Age of first pregnancy | -.119 | .249 | -.038 | -.478 | .633 | -.611 | .373 | -.093 | -.037 | -.035 | .850 | 1.177 |
| a. Dependent Variable: total | | | |  |  |  |  |  |  |  |  |  |  |

| **Collinearity Diagnostics^a^** | | | | | | | |  |  |
| --- | --- | --- | --- | --- | --- | --- | --- | --- | --- |
| Model | Dimension | Eigenvalue | Condition Index | Variance Proportions | | | |  |  |
|  |  |  |  | (Constant) | Number of children | Number of abortion | Age of first pregnancy |  |  |
| 1 | 1 | 3.905 | 1.000 | .00 | .02 | .02 | .00 |  |  |
|  | 2 | .894 | 2.090 | .00 | .02 | .01 | .00 |  |  |
|  | 3 | .709 | 2.347 | .00 | .03 | .90 | .00 |  |  |
|  | 4 | .277 | 3.755 | .00 | .63 | .03 | .00 |  |  |
|  | 5 | .199 | 4.433 | .02 | .26 | .05 | .07 |  |  |
|  | 6 | .016 | 15.433 | .98 | .05 | .00 | .92 |  |  |
| a. Dependent Variable: total | | | |  |  |  |  |  |  |
