# Supplementary material for: The association between reproductive history and menopausal symptoms: an evidence from the cross-sectional survey
Source: BMC Womens Health. 2022 Apr 27;22:136. doi: 10.1186/s12905-022-01715-z (PMC9044690; doi:10.1186/s12905-022-01715-z)

Supplementary file 2: outlier analysis.

| **Descriptives** | | | | |
| --- | --- | --- | --- | --- |
|  |  |  | Statistic | Std. Error |
| sum.j1.j11 | Mean | | 19.7430 | .41760 |
|  | 95% Confidence Interval for Mean | Lower Bound | 18.9198 |  |
|  |  | Upper Bound | 20.5661 |  |
|  | 5% Trimmed Mean | | 19.3899 |  |
|  | Median | | 19.0000 |  |
|  | Variance | | 37.319 |  |
|  | Std. Deviation | | 6.10890 |  |
|  | Minimum | | 11.00 |  |
|  | Maximum | | 38.00 |  |
|  | Range | | 27.00 |  |
|  | Interquartile Range | | 8.00 |  |
|  | Skewness | | .802 | .166 |
|  | Kurtosis | | .154 | .331 |
| sum.r12.r23 | Mean | | 22.0981 | .57275 |
|  | 95% Confidence Interval for Mean | Lower Bound | 20.9691 |  |
|  |  | Upper Bound | 23.2271 |  |
|  | 5% Trimmed Mean | | 21.6147 |  |
|  | Median | | 20.0000 |  |
|  | Variance | | 70.202 |  |
|  | Std. Deviation | | 8.37864 |  |
|  | Minimum | | 12.00 |  |
|  | Maximum | | 46.00 |  |
|  | Range | | 34.00 |  |
|  | Interquartile Range | | 13.00 |  |
|  | Skewness | | .826 | .166 |
|  | Kurtosis | | -.325 | .331 |
| sum.e24.e29 | Mean | | 11.4206 | .28522 |
|  | 95% Confidence Interval for Mean | Lower Bound | 10.8583 |  |
|  |  | Upper Bound | 11.9828 |  |
|  | 5% Trimmed Mean | | 11.2331 |  |
|  | Median | | 11.0000 |  |
|  | Variance | | 17.409 |  |
|  | Std. Deviation | | 4.17243 |  |
|  | Minimum | | 6.00 |  |
|  | Maximum | | 23.00 |  |
|  | Range | | 17.00 |  |
|  | Interquartile Range | | 7.00 |  |
|  | Skewness | | .431 | .166 |
|  | Kurtosis | | -.696 | .331 |
| sum.j1.e29 | Mean | | 53.2617 | 1.04017 |
|  | 95% Confidence Interval for Mean | Lower Bound | 51.2113 |  |
|  |  | Upper Bound | 55.3120 |  |
|  | 5% Trimmed Mean | | 52.6765 |  |
|  | Median | | 51.0000 |  |
|  | Variance | | 231.537 |  |
|  | Std. Deviation | | 1.52163E1 |  |
|  | Minimum | | 29.00 |  |
|  | Maximum | | 93.00 |  |
|  | Range | | 64.00 |  |
|  | Interquartile Range | | 21.25 |  |
|  | Skewness | | .588 | .166 |
|  | Kurtosis | | -.393 | .331 |

Boxplot of somatic symptoms:


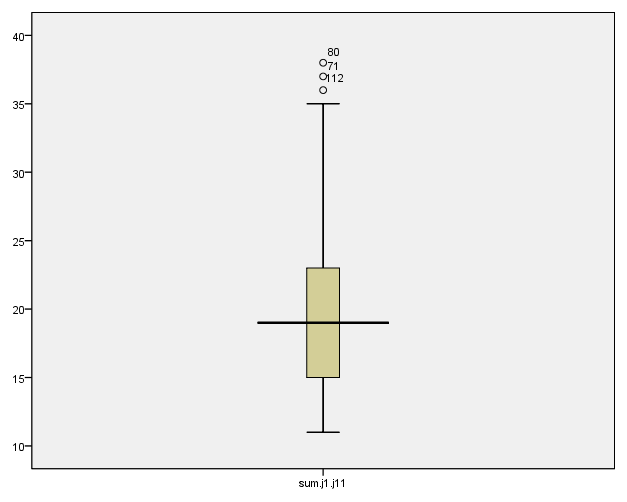


Boxplot of psychological symptoms:


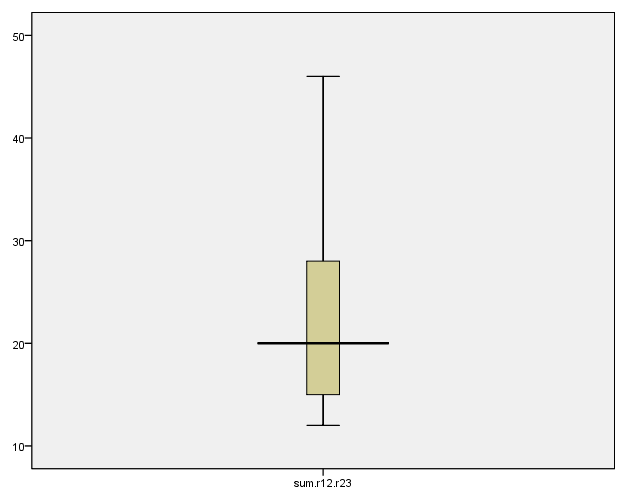


Boxplot of Urogenital symptoms:


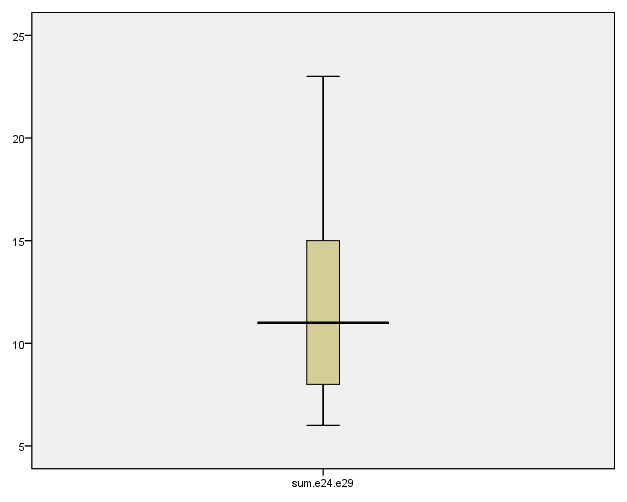


Boxplot of Total:


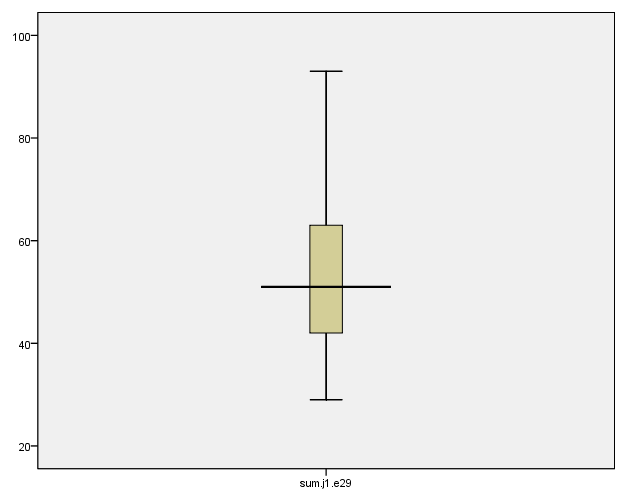

Supplement: Supplementary file 2 — Additional file 2. Outlier analysis. [file 12905_2022_1715_MOESM2_ESM.docx]
